# TAX and HBZ: hFc Ɣ 1 proteins as targets for passive immunotherapy 

**DOI:** 10.22038/IJBMS.2022.64787.14266

**Published:** 2022-05

**Authors:** Mohammad Mehdi Akbarin, Houshang Rafatpanah, Saman Soleimanpour, Abbas Ali Amini, Amirali Arian, Arman Mosavat, Seyed Abdolrahim Rezaee

**Affiliations:** 1 Inflammation and Inflammatory Diseases Research Center, Faculty of Medicine, Mashhad University of Medical Sciences, Mashhad, Iran; 2 Department of Microbiology and Virology, School of Medicine, Mashhad University of Medical Sciences, Mashhad, Iran; 3 Cancer and Immunology Research Center, Research Institute for Health Development, Kurdistan University of Medical Sciences, Sanandaj, Iran; 4 Animal Laboratory, School of Medicine, Mashhad University of Medical Sciences, Mashhad, Iran; 5 Blood Borne Infections Research Center, Academic Center for Education, Culture, and Research (ACECR), Razavi Khorasan, Mashhad, Iran

**Keywords:** ATLL, HBZ, HTLV, Immunization passive, Pichia pastoris, Recombinant proteins, TAX

## Abstract

**Objective(s):**

Human T leukemia virus type one (HTLV-1) causes two life-threatening diseases in around five percent of infected subjects, a T cell malignancy and a neurodegenerative disease. TAX and HBZ are the main virulence agents implicated in the manifestation of HTLV-1–associated diseases. Therefore, this study aims to produce these HTLV-1 factors as recombinant Fc fusion proteins to study the structures, their immunogenic properties as vaccines, and their capability to produce specific neutralization antibodies.

**Materials and Methods:**

TAX and HBZ sequences were chosen from the NCBI-nucleotide database, then designed as human Fc chimers and cloned into *Pichia pastoris*. Produced proteins were purified by HiTrap affinity chromatography and subcutaneously injected into rabbits. Rabbit Abs were purified by batch chromatography, and their neutralization activities for the HTLV-1-infected MT-2 cell line were assessed. Furthermore, the protective abilities of recombinant proteins were evaluated in Tax or HBZ immunized rabbits by MT-2 cell line inoculation and measurement of HTLV-1-proviral load.

**Results:**

Specific Abs against Tax and HBZ can eliminate 2 million MT-2 cells in 1/1000 dilution *in vitro*. In challenging assays, the immunization of the animals using Tax or HBZ had no protective activity as HTLV-1 PVL was still positive.

**Conclusion:**

The result suggests that recombinant TAX and HBZ: hFcγ1 proteins can produce a proper humoral immune response. Therefore, they could be considered a passive immunotherapy source for HTLV-1-associated diseases, while total TAX and HBZ proteins are unsuitable as HTLV-1 vaccine candidates.

## Introduction

In 1980 based on a Japanese report from a patient with T cell malignancy, the Human T cell leukemia virus (HTLV-1) was first described and belonged to the retroviridae family (1, 2). It has been estimated that worldwide HTLV-1 infection is around 10–20 million people (3, 4). HTLV-1 is endemic in several world regions, such as southwestern Japan, the Melanesian -basin, South America, Central Africa, the Caribbean Islands, and the Middle East, mostly northeastern Iran (3-5). HTLV-1causes two life-threatening diseases, adult T cell leukemia/lymphoma (ATLL) and HTLV-1-associated myelopathy/ tropical spastic paraparesis (HAM/TSP) in only 3–5% of infected subjects (6, 7). Thus the main proportion of HTLV-1infected individuals remains healthy carriers (2, 8). It is not completely understood why only a small percentage develop into the nominated associated diseases after a long time of infection (1). At the same time, the vast majority of HTLV-1 infected individuals stay on the asymptomatic side (9). Adult T cell leukemia/ lymphoma (ATLL) is infected clonal T-cell (CD4+, CD25+) progression, proliferation, and malignant transformation with a very poor prognosis (10). Moreover, cutaneous lesions, lymphadenopathy, hepatosplenomegaly, highly elevated blood levels of calcium and lactate dehydrogenase (LDH) are the main ATLL associated acute signs (10, 11). These outcomes happen in 2–5% of infected individuals after a long period of asymptomatic infection (12). A different range of organs such as skin, spleen, liver, lungs, and lymphoid glands are affected by tumor invasion (7, 10, 11). Furthermore, in the acute and lymphomatosis stages of ATLL, patients suffer from life-threatening opportunistic infection (11). The molecular events principle of ATLL is not entirely clarified, but the primary role of TAX and HBZ Onco proteins in ATLL pathogenesis is precisely confirmed (10, 13, 14). 

Like another retrovirus, HTLV genome comprises two positive sense single-strand RNAs, which contain gag, pol, and env, with two LTR regions on both sides and a pX regulatory unit nearby 3’LTR (15, 16). A 353 Amino Acid protein: Tax and 209 amino acid HBZ are the main protein virulence agents encoded by the pX region of HTLV-1(15, 17). These proteins undertake different post-translational modifications such as ubiquitination and phosphorylation, therefore organizing their subcellular and localization function is seen to be important (13, 16). HBZ and TAX are involved in transactivation, deregulation of apoptosis pathway, DNA repair distraction, genomic instability, and oncogenic transformation (13, 14, 18). However, Tax is extremely immunogenic and can be targeted by CTL-specific responses. Therefore, HTLV-1 has progressed with smart policies to firmly normalize Tax expression on the cell surface while preserving the source of transformation by HBZ accumulates expression (18-20)

Moreover, this process leads to generating a high genome integrated virus through infected cells, which then can accumulate viral oncoprotein(14). Finally, this cell cycle circulation produces an uncontrolled and aggressive proliferation within the infected T cells. The Tax oncoprotein applies a wide range of host cellular transcriptional mechanisms to control viral protein expression, such as NF-κB to make clonal expansion and proliferation of T cells for increasing the proviral load of HTLV (13, 21, 22). Besides the TAX protein down-regulation in ATLL T cell transformation, HBZ transcript from the minus strand 3′LTR up-regulate(13). Therefore, HTLV-1 can escape from cell-mediated immune responses (13, 23). HBZ in protein type can decrease transcription of the tax gene by the LTR region obstruction of CBP/p300 and ATF/CREB factors. HBZ increases transcription of CCR4, Foxp3, and T-cell immunoreceptors with Ig and ITIM domains (TIGIT) (23).

Moreover, HBZ also has activities in its RNA shape. HBZ RNA can lower apoptosis and increase infected T cells’ production (17, 19, 24). Because cytotoxic T cells cannot identify HBZ RNA, HTLV-1through this strategy is hidden from immune responses (25). HBZ plays a critical role in latent preserving infected T cells; therefore, these viral proteins could conduct malignant transformation.

Possible treatments for HTLV-1 infection are made by chemotherapy regimens, nucleoside/nucleotide reverse transcriptase inhibitors (NRTIs), Zidovudine (AZT), and Interferons (26, 27). However, no such treatments, even combination therapy, have been effective for HTLV-1-associated diseases. For example, the mean survival time for acute leukemic or lymphomatosis types of ATLL is around 5–11 months (26, 28, 29). On the other hand, from the time of HTLV-1 discovery, many attempts were conducted to develop an effective protective vaccine, which has yet to be successful (28, 30). Therefore, the newly discovered protein, HBZ, as a vital agent for HTLV-1 replication and inducing malignancy when TAX is down-regulated, may be a good candidate for vaccine development or the key target for passive immunotherapy.

Furthermore, direct antiviral antibodies have not been developed for HTLV-1 infection to test in passive immunotherapy (29, 31). Anti-Tax and anti- HBZ targeting the most important regulatory factors of HTLV-1 replication and inducing associated diseases should be the main target in such conditions. One of the important challenges in the immune response against certain proteins is the ability of immune cells to uptake antigens selectively (32). The antigen-presenting cell (APCs) via FcγRI can increase selective uptake of the Fc fragment of IgG (33). Therefore, using the Fc fragment of IgG1, it is possible to deliver the antigen of interest in the form of the recombinant Fc fusion protein to the APCs for inducing Th1 immune responses. Dendritic cells (DCs) and macrophages have multiple types of Fc receptor binding domain sites (FcγR), such as FcγRI, FcγRIIA, FcγRIIB, and FcγRIII, which can easily attach to the Fc-IgG domain (34).

Moreover, these domains boost antigen uptake, processing, and presentation 50–500 times for producing immune responses (33). Furthermore, Fc- recombinant proteins can increase solubility, half-life, and stability and assist in the purification process (33). Therefore, in this study, recombinant TAX-Fc and HBZ-Fc proteins were designed and produced to evaluate their protein structures and protective efficiencies and neutralize specific antibodies for passive treatment. 

## Materials and Methods

***Design and gene construction***

**Figure 1:**
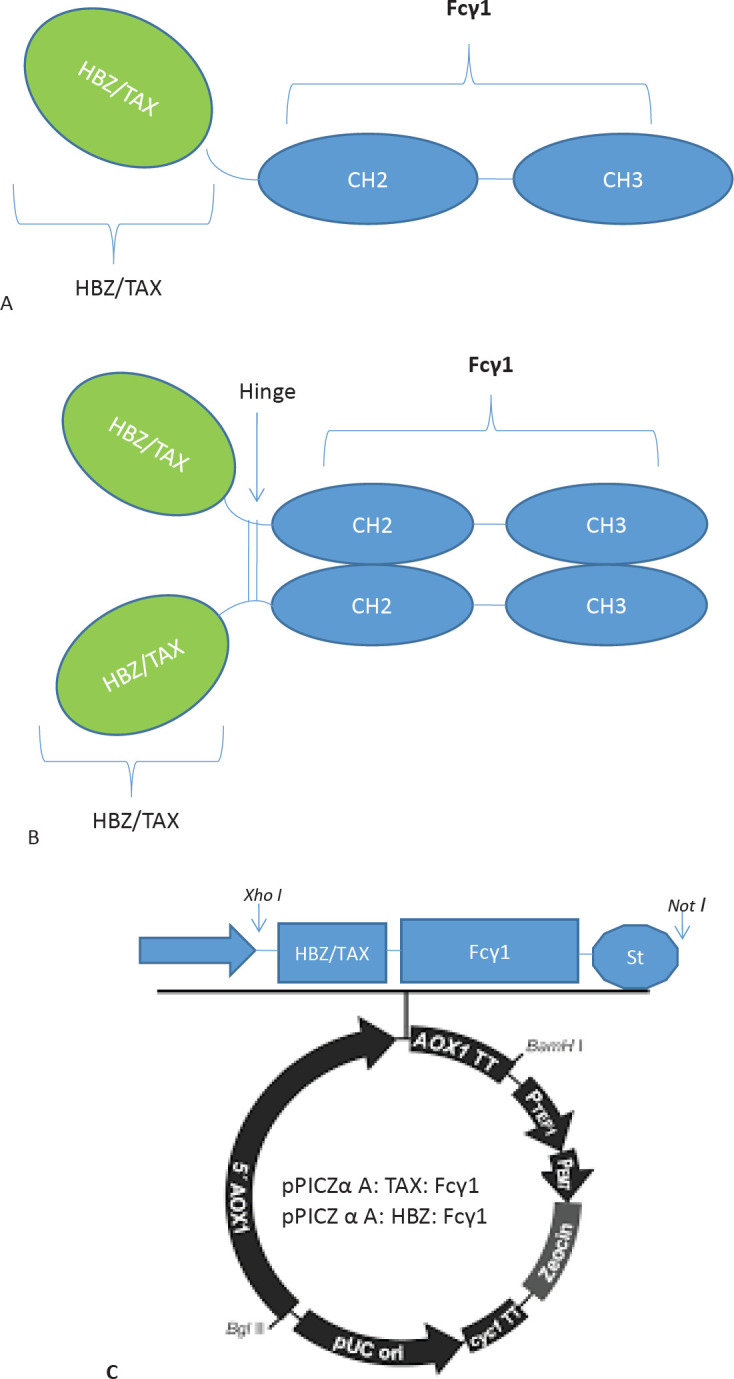
Schematic illustration of the Protein constructs. A) Schematic illustration of the monomer genetic fusion of HTLV fusion proteins TAX, HBZ, and Fcү1 to create a TAX: Fcү1 and HBZ: Fcү1 fusion protein. B) Schematic illustration of the genetic fusion of the dimmer genetic fusion of HTLV fusion proteins TAX, HBZ, and Fcү1 to create a TAX: Fcү1 and HBZ: Fcү1 fusion protein. C) Schematic map of pPICZα: TAX/HBZ:hFcү1. The insert was cloned into the XhoIand NotI restriction enzyme sites of the pPICZα vector downstream to the AOX1 promoter. 5′ AOX1, alcohol oxidase 1 promoter; AOX1 TT, transcriptional terminator from Pichia pastoris AOX1 gene; TEF1 promoter, transcriptional elongation factor 1 promoter from Saccharomyces cerevisiae; EM7 promoter, synthetic prokaryotic promoter; Zeocin, Zeocin resistance gene; CYC1 TT, transcriptional terminator from *Saccharomyces cerevisiae* CYC1 gene; pUCori, pUC origin of replication

For high-level expression of fusion proteins TAX: Fcγ1 and HBZ: Fcγ1, encoding sequences were optimized for *P. pastoris *(Figures 1A-B). The HBZ and TAX protein sequences were chosen from UniProt ID P0C746 and P03409. Gene runner (version 6) converted these protein constructions to DNA sequences. The genomic sequence of the hinge region, CH2, and CH3 domain of human IgG were selected from the NCBI Gen BANK database by Sequence ID: MK360906.1. After that, pUC57 cloning vectors were chosen to amplify optimized constructs. The restriction sites of enzymes (*Not*I and *Xho*I) were located upstream and downstream of the gene constructs, respectively. A series of three stop codons were placed at the end of each construct (Figure 1C). After sequence cloning, the constructs were extracted from the cloning vector by nominate restriction enzyme (Thermo Scientific, USA) and then transferred into the pPICZα A expression vector (Figure 1C). Lastly, recombinant vectors of pPICZα A-TAX: Fcγ1 and pPICZα A-HBZ: Fcγ1 were attained.

Furthermore, recombinant vectors were transferred to *Escherichia coli* Top10F’. For selection of transformed bacteria, the Laurie Bertani agar containing 25 μg Zeocin ™ was applied. The recombinant vectors were purified using the QIAGEN Plasmid Mega Kit (cat. 12181). In the end, both recombinant vectors were fully sequenced to ensure cloning accuracy. 

***Protein modeling of TAX and HBZ: Fcγ1a in silico***

*Molecular modeling of HBZ: Fcγ1 in silico*

Three-dimensional models of the HBZ: Fcγ1 sequences were constructed via homology modeling. BLAST sequence homology searches were performed to identify the template proteins. For modeling the protein, the HBZ 77 in complex with KIX and c-Myb(Protein Data Bank (PDB) entry: 6DNQ), crystal structure of Endophilin Bar Domain (PDB entry: 1X04), crystal structure of the CHIP U-box E3 ubiquitin ligase(PDB entry: 2C2L), and Fcγ1 (PDB entry: 1FC1) were chosen as the templates of HBZ and Fc domains, respectively. Model building was performed in the program MODELLER10.1 using a model-ligand algorithm (http://salilab.org/modeller/). Several models at various refinement levels were generated. Finally, the structures were minimized by the YSARA energy minimization server and Chimera 1.14 (35). All models were validated using ERRAT and PROCHECK at the University of California, Los Angeles (UCLA) (https://saves.mbi.ucla.edu/) (36, 37). Moreover, NetNGlyc 1.0 Server(http://www.cbs.dtu.dk/services/NetNGlyc/) and NetOGlyc 4.0 Server (http://www.cbs.dtu.dk/services/NetOGlyc/) were applied to determine N- and O-glycosylation sites(38, 39).

*Molecular modeling of TAX: Fcγ1 in silico*

Based on lack of a similar model for TAX protein, it was modeled by I-TASSER (Iterative Threading ASSEmbly Refinement) and an online modeling site (https://zhanggroup.org/I-TASSER/). Next, homology modeling was applied for recombinant TAX: Fcγ1 construct by using Fcγ1 (PDB entry: 1FC1) as the template of Fc domains. Buildings of the probable model were done in the program MODELLER10.1 using a model-ligand algorithm (http://salilab.org/modeller/). Several models at various refinement levels were generated. Finally, the YSARA energy minimization servers and Chimera 1.14 were used to attain stable nearby minimum energy of protein structure(35). All output models were validated using the programs ERRAT and PROCHECK at the University of California, Los Angeles (UCLA) (https://saves.mbi.ucla.edu/) (35, 36). Moreover, N- and O-glycosylation sites were identified by NetNGlyc 1.0 Server (http://www.cbs.dtu.dk/services/NetNGlyc/) and NetOGlyc 4.0 Server (http://www.cbs.dtu.dk/services/NetOGlyc/)(38, 39).

***Subcloning to p. pastoris***

After plasmid extraction, recombinant vectors were cleaved by SacI enzyme to linearize. The linearized engineered vectors were electroporated to transfer into *P. pastoris *GS115 cells. YPD agar containing 100-1000 µg Zeocin™ (InvivoGen, USA) was used to select the transformed *P. pastoris *GS115 cells from non-transformed cells after growth under 3 days of incubation at 28 °C. The Zeocin™ resistance colonies could be grown on YPD agar and demonstrate the correction of expression vector transmission.

***Expression at a low scale and detection of recombinant protein***

Confirmed transformed yeast cells were cultured in a 25 ml baffled flask containing 5 ml BMGY (28 °C in shaker incubator) to reach OD_600_ = 2 (about 16–24 hr) to recognize the top colony expressing recombinant proteins. After getting the desired OD 2-6 at 600 nanometers, the yeast cells were collected with centrifugation (5000 rpm for 5 min at 4 °C) and then cultured in BMMY to reach OD_600_=1. For stimulation, the expression of recombinant proteins methanol was added in the final concentration of 0.5% into the BMMY flasks and kept at 28 °C for 5 days (within a shaker incubator with 300 rpm). The methanol concentration was kept at the optimum amount by adding 100% alcohol to each baffled flask daily. Finally, the supernatant was obtained by centrifugation (11000 rpm for 10 min at 4 °C), and the expression amount of total and recombinant proteins was determined by the pyrogallol red method, Bicinchoninic Acid method (BCA), and enzyme-linked immunosorbent assay (ELISA), respectively. According to those data, the optimal protein-expressing colonies were selected.

***Larg-scale expression of recombinant proteins***

The optimization was conducted as previously described to start high-level production of the recombinant Fc fusion proteins. After that, 400 ml of BMGY was divided into two 200 ml bottles, and the best colonies were inoculated within the media base on ELISA results. Then each bottle was incubated at 28 °C and shaken at 300 rpm to reach OD_600_= 6. After that, the cultured yeast cells were acquired by centrifuge (5 min at 4 °C and 5000 rpm) and re-cultured in four 300 ml BMMY mediums. Therefore, the final concentration was equal to OD_600_=2. The baffled flasks were incubated at 28 °C for 3 days and shaken at 300 rpm. Then 100% methanol was added to each flask at a final concentration of 1% every 24 hr exactly to stimulate protein expression. 

***Purification of recombinant proteins***

For recombinant protein purification, the supernatant of the BMMY was obtained with centrifugation (12 min at 10,000 rpm and 4 °C), then filtered by 0.45 Millipore filters. Then the recombinant proteins were purified by the HiTrapr Protein A/G Sepharose Fast Flow column (GE Healthcare, USA) as previously described (40, 41). The pH of the eluted protein solution was rapidly neutralized by adding the proper amount of neutralization buffer (1M Tris-HCl, pH 9). For changing the buffer of eluted proteins to PBS, the protein fractions were transferred to the Amicon® membrane 10kDa and then centrifuged for 10 min at 4 °C and 12000 rpm. 

***Assessment of protein concentration***

Protein concentration was measured by the pyrogallol red method (PRM) Bio labo-France (Cat: 97016). The first amount of 1 ml from low scale media was centrifuged in a 1.5 ml microtube at 5000 rpm, and then the supernatant was tested by auto analyzer BA 400 Biosystem for total protein. To measure purified protein concentration, the bicinchoninic acid assay (BCA) method was applied by Pars Toos assay kit (Cat: A101251). Working solution was prepared to examine the protein concentration by adding 1 part of Copper Reagent to 50 parts of BCA Reagent. Then 75 μl of each sample and standard were added to each 96 well microplate beside the 250 μl of working reagent, shaken by small tapping, and incubated at 37 °C for one hour. A serial dilution of BSA protein from 1000 μg/ml to 1 μg/ml was created, and the concentration of each purified protein was calculated based on standard OD in 562 nm. 

***Recombinant protein detection by blotting***

Two blotting dot and soft gel methods were applied to detect the TAX: Fcγ1 and HBZ: Fcγ1 protein. A suitable piece of PVDF membrane was obtained for dot blotting assay, and grid drawing was done with a pencil. PVDF membrane was pre-incubated with absolute methanol for 30 sec for activation and then washed with PBS buffer (pH 7.5). 5 µl of each sample was slowly pipetted onto this membrane in the appropriate square. After that, the membrane was let to dry for 15 min at room temperature. Using 5% skim milk /PBS buffer as the blocking reagent incubation for one hour at 37 °C or overnight at 4 °C is performed. The papers were washed three to five times with PBS buffer and incubated with anti-human HRP serial dilutions (1/1000 to 1/5000) for one hour at 37 °C by shaking on the rocker. 1-1.5 ml of BCIP was added (5-Bromo-4-chloro-3-indolyl phosphate), further reacted with NBT (nitro blue tetrazolium) as substrate, then incubated 15 min at RT, and washed two times by PBS buffer (pH 7.5). The soft gel dot blotting was applied to select the best colony of recombinant protein production. For this method, three pieces of PVDF paper were activated by absolute methanol for 30 sec and located on the sterile polyperen plate. Candidate colony from YPDS agar was selected and added into the 10 ml BMMY medium with 2 g agar powder gently mixed and cooled to 30–40 °C, vortexed rapidly, and filled into the three parts of the plate. The plate was incubated at 22 °C for 48 hr, the gel separated, the PVDF papers washed with PBS and then the paper blotted as in a previous method. This method can rapidly predict an Fc recombinant protein production and determine the best-transferred colony.

***Confirmation of Fc tag protein production by ELISA***

The ELISA homemade method was designed using a microplate immune sensitized polystyrene surface to assess recombinant protein production. Briefly, 100 microliters of coupling buffer (carbonate/bicarbonate buffer at pH>9) was mixed gently with 100 μl of protein solution and added to each well, incubated for 1 hr at 37 °C or overnight at 4 °C. Anti-human IgG was used as probe and TMB as substrate. Finally, the enzymatic reactions were stopped by sulfuric acid (0.1 N). The concentrations of Fc tag proteins were measured in OD 450 and 620 as reference wavelengths using a Hyperion microplate reader (USA).

***SDS-PAGE and Western blot***

SDS-PAGE and Western blotting were obtained to analyze recombinant proteins’ molecular weight and expression. Polyacrylamide gel in 12% concentration was used to separate the recombinant protein by electrophoretic force Bio-Rad Mini PROTEAN electrophoresis (Bio-Rad, USA), and then the gels were stained by Coomassie Brilliant Blue G-250. After separating the protein, the Western blotting technique was obtained to prove the Fc-recombinant protein expression. For this purpose, the proteins were transferred onto polyvinylidene fluoride (PVDF) membrane by electro transferring, and BSA 2% was used to block PVDF membranes (1 hr at 37 °C). Then the PVDF membranes were washed three times with PBS and incubated with goat anti-human IgG-HRP antibody (Santa Cruz, USA) at a dilution of 1:3000 for 1 hr at room temperature. The incubated membrane was washed similar to the previous step, and Fc-tag recombinant protein (TAX and HBZ: hFcγ1) was identified by Nitrobluetetrazoliumchloride/5-Bromo-4-chloro-3- indolyl phosphate (NBT/BCIP).

***APC-targeting of TAX and HBZ: Fcγ2a recombinant protein***

A direct immunofluorescence assay was used to confirm the Fc receptor attachment ability of Fc-recombinant protein. Briefly, the PBMCs were obtained from the peripheral blood of a humane donor by the ficoll gradient method. Then a slide smear was prepared and fixed, and then incubated at 37 °C for an hour by recombinant proteins. Each slide was washed with PBS solution and incubated with PE anti-human CD64 (FcγRI) (BioLegend, USA) and goat anti-human IgG1-FITC (Santa Cruz, USA) in 3% (w/v) BSA in a humidified chamber for 2 hr. The microscopic evaluation was done by a fluorescence microscope (Nikon Eclipse E200, Japan), and proper images were selected.

***Rabbit immunization by recombinant proteins***

*Preparation of the adjuvant compound and recombinant protein*

The appropriate concentrations of Fc-fusion proteins (100 µg of each protein) were obtained by dilution of sterile PBS in a 1.5 ml microtube. These microtubes were mixed vigorously for 30 min with an equal volume of complete and incomplete Freund adjuvant for the first and second injections, respectively (the final concentration was 50 µg at 25 microliters). Each volume was used for injection with a sterile 23 gauge syringe needle.

***Polyclonal antibody production***

For polyclonal antibody production, two female New Zealand white rabbits were used at six weeks and at least 2 kg weight for each recombinant protein. The 50 µg in 25 µl volume of desired proteins were injected subcutaneously into the back of the rabbit neck for a 2-week interval up to three times. Each rabbit was fed specific pet food and carrot three times a week until the end of immunization. Two weeks after the last injection, a 5–7 ml whole blood sample was obtained after using an analgesic/sedative combination (butorphanol, 0.2 mg/kg; acepromazine, 0.1 mg/kg) into the clot activator vacuum tube. After the collected blood was clotted, the serum was separated by centrifuge at 3500 rpm for 5 min. The collected serum was used for separating the polyclonal antibody by recombinant protein batch chromatography.

*Recombinant protein batch chromatography*

To prepare ligand protein, 500 μl of TAX and HBZ protein solution (750 μg) with an equal volume of coupling buffer (pH 8.3) was mixed and incubated overnight at 4 °C. The sepharose beads were activated in 20–50 ml of 1 mM cold HCl for 15 min at 4 °C, and then the activated beads were washed with 1 mM HCl. The activated beads were incubated with desired proteins as ligand overnight at 4 °C. The sepharose beads were washed by coupling buffer and blocked by 5 ml 0.1 M Tris-HCl buffer (pH 8.0) for 2 hr at room temperature. The beads were washed at least three cycles with acid and alkali buffer alternatives (0.1 M acetic/sodium acetate, 0.5 M NaCl, pH4.0; 0.1 M Tris-HCl, 0.5 NaCl, and pH 8.0). Then, the beads were incubated with serum for 1–2 hr at room temperature. PBS buffer was applied for washing the beads. Therefore, non-attached antibodies were removed. Elution buffer (pH 2.5) was used for releasing the coupled polyclonal antibodies and neutralized immediately by saturated phosphate buffer at pH 7. The concentration of collected antibodies was calculated by the BCA method and then stored at -20 °C for more evaluations. 

***Antibody cell cytotoxicity assays***

For evaluation of infected malignant cell cytotoxicity, an MT-2 cell line (08081401Uk) was used. These cells were first thawed rapidly and then incubated in a 25 ml sterile flask by 5 ml RPMI with 10% FBS(Sigma) at 37 °C, 5% Co2, for 3–5 days. After cell recovery and proper proliferation, the cells were washed with PBS buffer (pH 7.5) and resolved for an appropriate count of 2 million/ml. Terasaki plates were obtained, and one µl of cell suspension was added to each well. The purified antibodies were added to each well of MT-2 cells and incubated at 37 °C for one hour. The control rabbit serum and anti HLA were used for negative and positive controls, respectively, then the 5 µl rabbit complement was added to each well. Following incubation for 1 hr at 37 °C by rabbit complement, the eosin dye was added to all wells and incubated at RT for 15 min. A Motic inverted microscope was used to examine the viability of cells for all wells, and cell death of more than 50 percent was considered positive. 

***Fc fusion recombinant protein immune protection assessment for viral transmission***

To evaluate the effect of our two recombinant proteins in protecting viral transmission, two weeks after the last subcutaneous injection of all immunization rabbits, each one was peritoneally inoculated with HTLV-1 by 2 million MT-2 cell lines. One rabbit of the same age, weight, and gender was used as a control sample. Three weeks after inoculation, PBMC cells were collected from the peripheral blood of each infected rabbit by the ficoll gradient method (Lymphodex, Inno-train, Germany). TriPure™ isolation reagent solution was applied for DNA extraction and the DNA concentration from all samples was examined and normalized by a nanodrop spectrophotometer. Persistence of the HTLV-1 proviral genome was investigated by HTLV-1proviral real-time PCR assay (Novin gen, Iran). 

## Results

***Molecular modeling of TAX and HBZ: hFcγ1 recombinant protein***

*Molecular modeling of HBZ: Fcγ1 recombinant protein*

**Figure 2:**
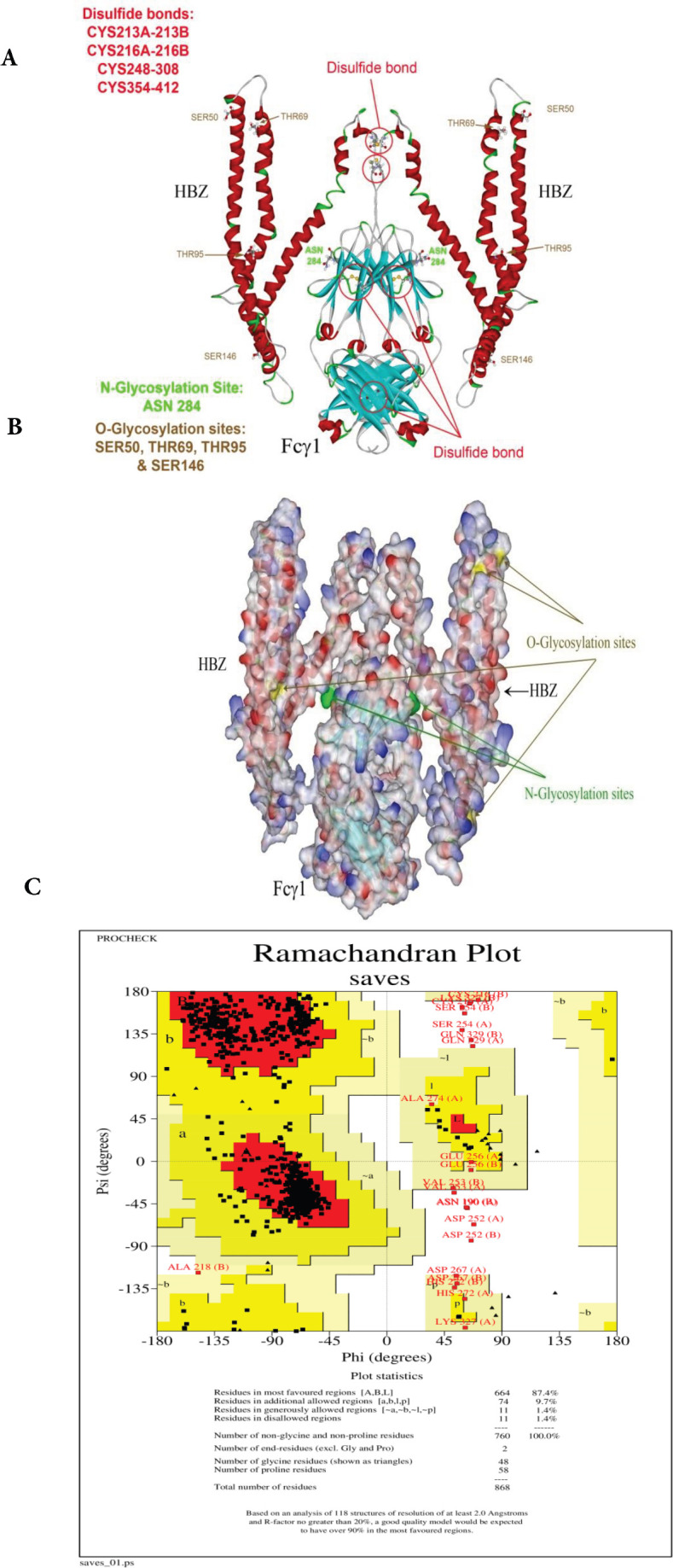
Protein modeling results for HBZ: hFcγ1. A) Solid ribbon views of the modeled protein, which include the dimeric form of Fc tag and HBZ. The red circle identifies the disulfide bond, and the amino acid residues were nominated. B) Both N and O glycosylation sites were displayed in yellow and green colors. The N glycosylation sites are located on Fc taq, while the O sites carry HBZ proteins. In dimeric form a total of 10 sites are available for glycosylation. C) The Ramachandran plot of the modeled protein displayed that 87.4% of whole recombinant proteins are located in the selected region. There are only 1.4% of residues located in the disallowed region

Primarily, Modeller software program was used to generate around 10 models, and one of them with the best ERRAT score was chosen for construction modifications. The dimeric form of recombinant HBZ protein was modeled, in which the disulfide bond was added between Cys213A-B, Cys216A-B, Cys 248-308, and Cys 354-412 for HBZ: hFcγ1 protein. The molecular procedure was performed for the geometry optimization form of the adapted dimeric assemblies proteins modeled (Figure 2a). HBZ is glycosylated in ASN 284 for N format and SER 50, THR 69, THR 95, and SER 146 for O format (Figures 2a and b). Solid ribbon and solvent surface presentations of the last computational model were summarized in Figures 2a and 2b. The ERRAT score for the final model was 89.11 %. The Ramachandran plot established a satisfactory assembly of designed proteins by confirming more than 87.4 % of residues in the preferred and allowable regions (Figure 2c).

*Molecular modeling of TAX: Fcγ1 recombinant protein*

**Figure 3:**
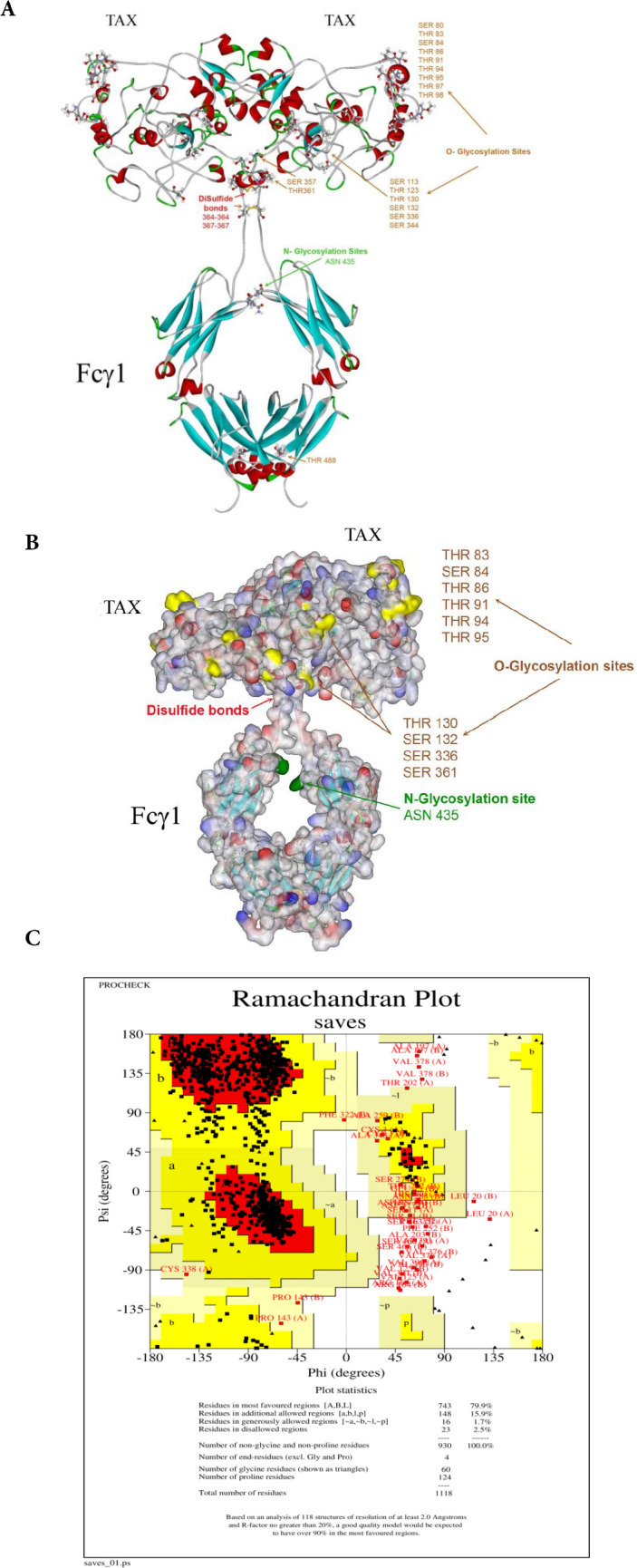
Protein modeling results for TAX: hFcγ1. A) Solid ribbon views of the modeled protein, which include the dimeric form of Fc tag and TAX. The red circle identifies the disulfide bond, and the amino acid residues were nominated. B) Both N and O glycosylation sites were displayed in yellow and green colors. The N glycosylation sites are located on Fc taq, while the O sites are carried on TAX proteins. In dimeric form a total of 13 sites are available for glycosylation. C) The Ramachandran plot of the modeled protein displayed that 79.9 % of whole recombinant proteins are located in the select region. There are only 2.5 % of residues located in the disallowed region

The modeling of *TAX: Fcγ1*was performed as described for HBZ: hFcγ1. The final model’s solvent surface and solid ribbon presentations were outlined in Figures 3 A and B. The final TAX model with Fc-tag had an ERRAT score of 88.89 (Figure 3-D). TAX is a highly glycosylated protein with several O-glycosylation sites and contains amino acids SER/THR80, 83, 84, 86, 91, 94, 95, 97, 98, 113, 123, 130, and 336. For N in the N-glycosylation site, there is just one ASN amino acid in position 435. The dimeric form of recombinant TAX protein was modeled, in which the disulfide bond was added between Cys 338-341, Cys 373-433, and Cys 479-537 for TAX: hFcγ1 protein. The Ramachandran plot established a satisfactory assembly of designed proteins by confirming more than 79.9 % of residues in the preferred and allowable regions (Figure 3c).

***Transformation and selection of the transforms***

After transformation by electroporation, the positively transformed colonies were selected by the YPDS medium containing 100 μg/ml Zeocin™. The Antibiotic selection serial dilution (100-1000 μg/ml) was done to choose the best-transformed colony. The best colonies were obtained from a 500 μg/ml concentration of Zeocin™.

***Soft gel and dot blotting results for confirming Fc recombinant protein***

**Figure 4:**
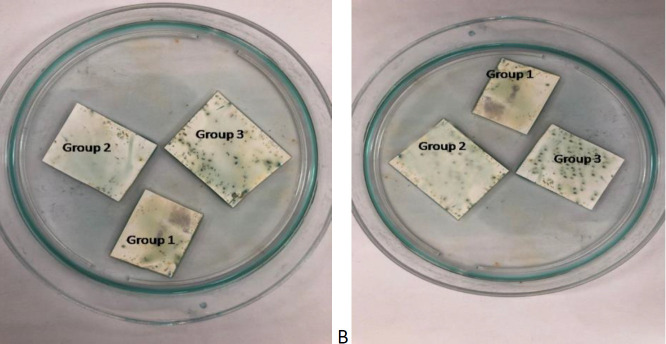
A Soft gel dot blotting for detection of TAX and HBZ: hFcγ1 optimum colonies. A) results suggest group number 3 (17, 18, 19) for Tax and B) (17, 19) for HBZ proteins have stronger transformed colonies

Soft gel dot blotting was performed to detect the best productive colony of TAX and HBZ: hFcγ1 (Figures 4 a-b). The observed colonies were divided into three groups: 1, 2, 8 cubes mixed for group one, 4, 10, 15 for group two, and 17, 18, 19 for group three for TAX: hFcγ1. For HBZ Fc tag recombinant protein, the three groups were introduced by cubes numbered 1, 2, and 4 as group one, 12 and 13 as group two, and 17 and19 as group three. 

**Figure 5:**
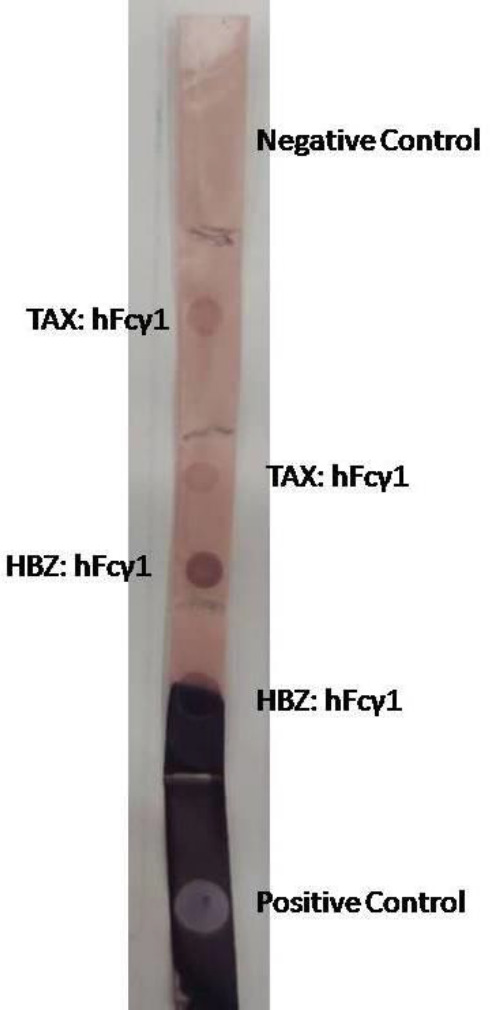
Dot blotting for detection of TAX and HBZ: hFcγ1 recombinant proteins and optimization of HRP antibody titration for the ELISA method. Our results demonstrate that titration of 1/3000 for HRP anti-human can have the best resolution

These results indicate that the number of transformed colonies can produce the desired protein more strongly than others; therefore, the dot blotting method was applied to determine the concentration of antibodies used in the ELISA method. The dot blotting results of proteins demonstrate that the 1/3000 titer of HRP conjugated anti-human is proper for detecting proteins by the ELISA method (Figure 5). 

***Optimization of recombinant fusion protein production***

PRM, on a low scale, was used for protein production assessments. Moreover, based on the PRM method, the optimum methanol concentration and incubation time were selected for high-scale protein production. THE PRM results for protein concentration were 36 mg/dl and 45mg/dl for TAX and HBZ: hFcγ1respectively, in 1% methanol on day 2 (Table 1). 

***Recombinant fusion protein purification and identification***

The Hi-Trap purified protein concentrations were 0.548 mg/dl and 0.867 mg/dl for TAX: hFcγ1 and HBZ: hFcγ1, respectively. The ELISA test was performed for these fractions which indicates the concentration of TAX: hFcγ1 as 0.509 mg/dl and for HBZ: hFcγ1 as 0.712 mg/dl (Table 2). These results are in coordination with the BCA protein assay for extracted eluted proteins. 

**Figure 6:**
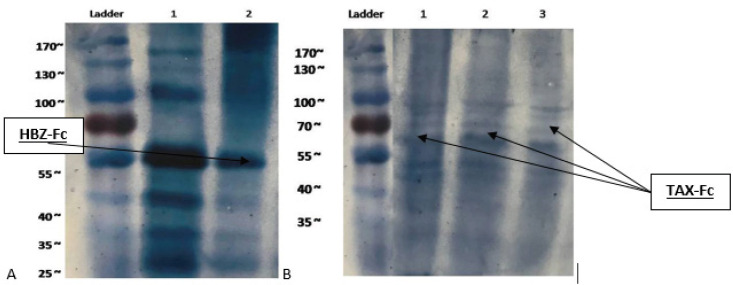
Analysis of the recombinant HBZ: hFcγ1 (a) and TAX: hFcγ1 (b) protein by 12 % SDS-PAGE stained with Coomassie Blue. A) Line 1 IVIG 1/100 and 2 HBZ: hFcγ1. B) Line 1, 2, and 3 TAX: hFcγ1, fractions of the eluted recombinant protein of approximately 51 and 65.5 kDa for HBZ and TAX: hFcγ1, respectively. Line Ladder: protein marker

 For Fc-tagged recombinant protein analysis and identification of purified fractions, SDS-PAGE was done then results were confirmed by western blotting assay. Our results demonstrate a 51 and 65 kDa protein band for HBZ and TAX, respectively ​(Figures 6A and B).

**Figure 7:**
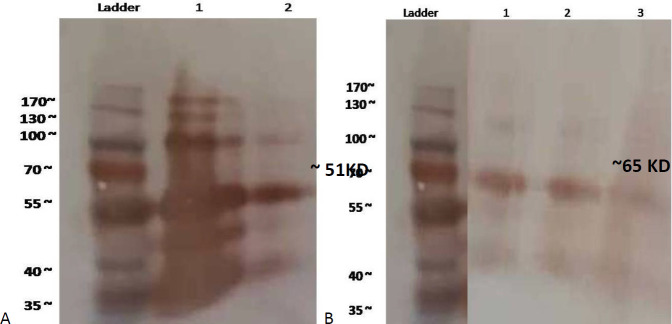
Expression of recombinant HBZ: hFcγ1 and TAX: hFcγ1 protein was analyzed by immunoblot using anti-Human IgG-HRP. A) HBZ: hFcγ1 identification by NBT/BCIP substrate line 1: IVIG, 2: HBZ: hFcγ1. B) TAX: hFcγ1 fraction protein blotting by NBT/BCIP substrate line 1, 2, 3 TAX: hFcγ1. Ladder: Protein marker 10-180 kDa

Western blot analysis established Fc tag recombinant protein production in predicted molecular weight, while for TAX: hFcγ1, variant glycosylation sites were identified more in the SDS page and western blotting assay than in HBZ: hFcγ1 (Figures 7A and B). 

***APC-targeting of FC recombinant fusion protein***

**Figure 8:**
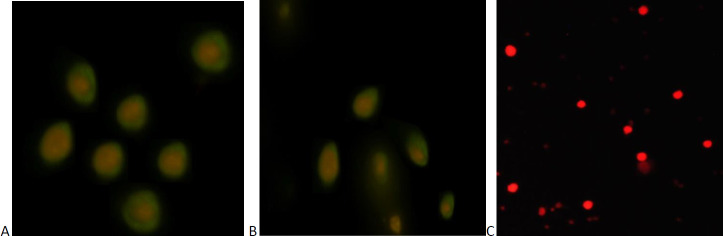
Co-localization of FcγRI (CD64) on human PBMCs and TAX/HBZ: hFcγ1 recombinant fusion protein. Immunofluorescence staining of PBMCs showing TAX: hFcγ1 and HBZ: hFcγ1 recombinant fusion binds to FcγRI A and B, respectively. Red signal, PBMCs stained with PE anti-human CD64 (FcγRI) antibody; green signal, TAX: hFcγ1and HBZ: hFcγ1 recombinant fusion stained with goat anti-human IgG1-FITC antibody. C) Immunofluorescence staining of PBMCs with goat anti-human IgG1-FITC, PE anti-mouse CD64 (FcγRI) without Fc-fusion protein as a negative control (red signal, PBMCs stained with PE anti-human CD64 (FcγRI) antibody). Fcγ1, Fc fragment of human IgG1; FcγRI, Fcγ receptor I; PE, phycoerythrin; FITC, fluorescein isothiocyanate

To demonstrate Fc-tagged recombinant protein HBZ: hFcγ1and TAX: hFcγ1 binding to Fcγ receptor (FcγRI) on APCs, a direct immunofluorescence assay was performed. Therefore, it shows the binding of Fc tag recombinant protein to the FcγRI on human PBMCs (Figure 8). In this method, attachment of hFcү 1 fusion protein to CD 64 co stain by red and green signal; therefore, the yellowish-green ring indicates peripheral binding to FcγRI on PBMCs. 

***Polyclonal antibody production values***

**Figure 9:**
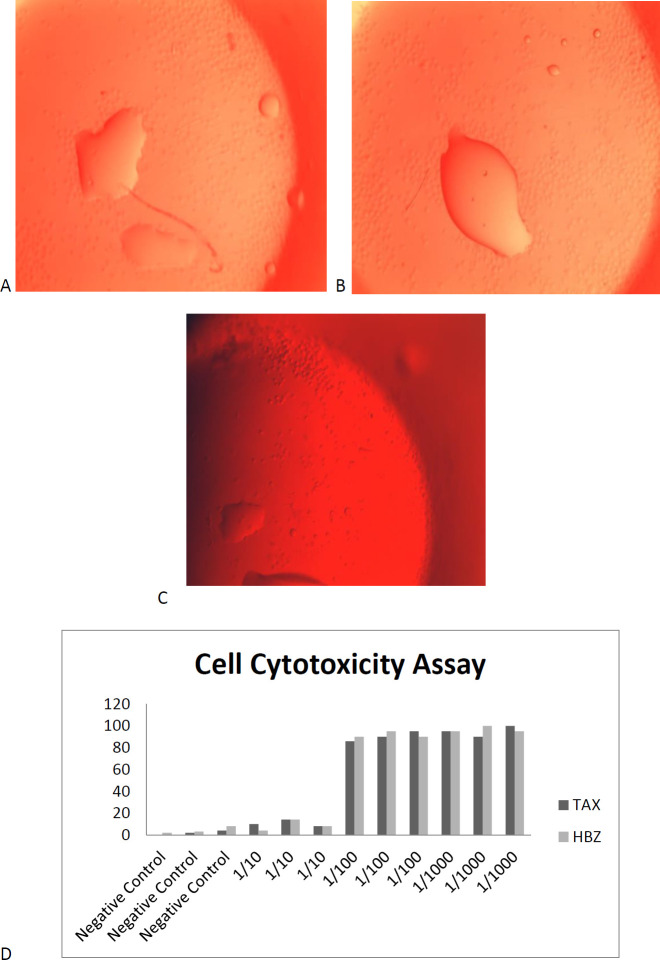
A) Cell cytotoxicity assays (CCA) were done by Anti-TAX: hFcγ. B) CCA results for Anti-HBZ: hFcγ in 1/1000 titration. C) The negative control contained MT-2 cells plus normal saline. D) CCA demonstrates polyclonal specific antibody (1-1/1000 titration) against TAX:hFcγ1 and HBZ: hFcγ1 recombinant fusion protein to the evaluated killing ability of around 2 million HTLV malignant cell line. The CCA display 1/1000 titer of PCA for both TAX, and HBZ: hFcγ has the absolute ability to neutralize 2 million MT-2 cells

5 ml of whole blood was obtained into the clot activator from each immuno sensitized rabbit. Two rabbits for each recombinant protein were applied for polyclonal antibody production. Serum was separated by 3500 rpm for 5 min at RT, then a specific polyclonal antibody was selected by batch chromatography. The concentrations of eluted antibodies were 124 mg/dl and 260.78 mg/dl based on the BCA method for TAX and HBZ, respectively. For achievement, the neutralizing properties of antibody cell cytotoxicity assay were done. The best titration of obtained antibodies for neutralizing 2 million MT-2 cell lines was 1/1000 for both recombinant proteins (Figure 9).

***Viral transmission evaluation in TAX and HBZ - Fc recombinant immune sensitized rabbits***

**Figure 10:**
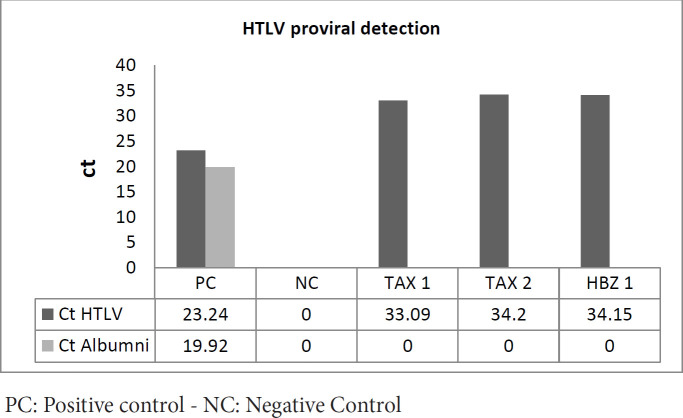
HTVL-I proviral examination by Real-Time PCR Tag-Man method. The human albumin is just presented for positive control, and none of the study groups were positive. The concentration of rabbit DNA was normalized around 60–70 ng/μl. The negative results for human albumin indicate the HTLV-1 genome detection results from inoculation, not the persistence of MT-2 cell lines

To evaluate the protective effect of TAX and HBZ: Fc proteins, viral infections were done using HTLV-1 infected MT-2 cell line injections. The protective effect of these Fc fusion proteins was examined by evaluating the HTLV-1 PVL in the experimental groups. The case group included 2 rabbits for TAX and one for HBZ (one of the rabbits in the HBZ group expired before inoculation of spastic paralysis) and a non-Fc fusion protein manipulated rabbit for control. The proviral load investigations demonstrate persistence of the HTLV-1 genome in the case group, while the control rabbit was not polluted with the MT-2 cell line (Figure 10). 

**Table 1:** Total protein concentrations of recombinant low-scale production were done by the Pyrogelal red method on the second day, and 1% concentration was the optimum protein production condition

Methanol %	Day 1		Day 2		Day 3		Day 4		Day 5		Day6		Day 7	
	H* mg/dl	T! mg/dl	H	T	H	T	H	T	H	T	H	T	H	T
0.5 %	16.8!	13.1	34.9	23.4	31.7	19.8	21.7	16.3	17.5	14.7	14.9	11.8	10.4	6.9
1%	26.7	17.4	53.6	38.3	41	29.5	24.6	21.4	18.2	18.9	15.7	14.5	12.7	10.2
2%	24.5	14.5	48.2	36	35.1	24	23.6	17.7	15.2	16.4	13.5	12.3	9.5	6

*H=HBZ: hFcγ1

! T=TAX: hFcγ1

**Table 2:** ELISA results for TAX and HBZ: hFcγ1 in comparison with serial dilution of IVIG 5-500 mg/dl. Our results demonstrate the concentration of HBZ as 0.712 mg/dl and 0.509 mg/dl for TAX: hFcγ1

Name of protein	OD	Concentration
HBZ-Fc	2.395	711.987
TAX-Fc	1.760	509.871
Control Negative	0.012	0
S-1	0.125	5
S-2	0.373	50
S-3	1,72	500

## Discussion

Although HTLV-1 is a non-pathogen in most infected individuals, the two types of associated diseases can induce serious problems for patients (42). Therefore HTLV infections can mostly be considered a public health problem in an endemic area. Molecular studies have clarified the role of two main proteins, TAX and HBZ, in the pathogenesis of the virus (13, 19). These proteins promote the pathogenesis of HTLV through the two major pathways: manipulation of CTL responses in HAM/TSP patients or malignant transformation in infected T cells (43, 44). Besides TAX and HBZ proteins, other regulatory agents encoded by the 3’ LTR region, such as p13, P 30, and HTLV protease, have a supportive role in HTLV pathogenesis (14). There were different strategies designed for HTLV-1 infection treatment, which could be divided into three main categories: antiviral treatment, immune activation treatment, chemotherapeutics regimes, and a combination of them (29, 45, 46). Marino-Merlo et al. in 2020 reviewed the antiviral treatments applied for HTLV infection in HAM/TSP and ATLL (29). Zidovudine-AZT (3’-azido-3’-deoxythymidine; AZT) was used in combination with anti-inflammatory drugs such as Danazol, nucleoside/nucleotide reverse transcriptase inhibitors (NRTIs) such as didanosine (2′, 3′-dideoxyinosine; ddI) and epigenetic regulator drugs such as valproic acid (VPA) for HAM/TSP patients (29). These therapeutic regimes have encountered problems such as genomic alteration of HTLV-1, lack of intracellular concentration of the active substance, and viral genome integrity; therefore, they could not be completely successful (27, 29).

Furthermore, the efficiency of VPA was examined by Olindo et al. during an open-label trial for two years. They observed that the HTLV-1proviral load and CTL function were not affected by VPA usage (47). In 1995 AZT was firstly used in association with interferon-alpha (IFNa) for treatment of ATLL and an overall function was demonstrated in increasing the survival period of patients (26). However, these results did not bring better outputs than chemotherapy treatments(29). Moreover, no nominated treatments can completely cure ATLL individuals. Some combinations of antiviral treatment with bone marrow transplantation diminish the clinical compilations but cannot control virus spread (46). 

Immune response against HTLV-1 was considered a potential source for treatment for several decades. The HTLV vaccine was first described by de Thé et al. in 1993, which suggests the envelope (Env) antigen, and the results were successful in the animal model scale (30). These studies were followed three years later by both env and gag coding sequence recombinant proteins. One of the biggest challenges was producing neutralizing antibodies and specific cell-mediated immunity (48). They recommend using their proteins for high-risk HTLV population transmission control. Several studies suggest using peptides such as Tax, p12, p13, p30, and p24 for HTLV immunization and vaccine development (15, 49). In 2019 it was demonstrated that most proteins of HTLV have conserved combinations within infection and replication, while their sequence variation is less than 3 present in the whole genome (15). Based on the conserved sequence and antigen properties, these results suggest using HTLV protein epitopes to induce an immune response. As we observed, one of the rabbits died because of spastic paralysis in the HBZ group. The results of the proviral assessment demonstrate the case rabbits are more susceptible to infection than normal control suggested. TAX/HBZ recombinant protein may have a pathogenic function in the host (Figure 10). Three main questions about this event are possible: one is how these Fc fusion pure proteins have functioned within the cells without infection? The possible pathway for outer to inner cell transfer is the FcRn receptor on subcutaneous APC cells such as dendritic cells and macrophages (50, 51). These receptors led to the transfer of Fc fusion protein from the outside of the cell wall to the cytoplasm to conserve the fusion protein structure(51). Another possible question is about the role of TAX and HBZ in changing hosts susceptible to infection? Previous studies demonstrate the direct role of TAX and HBZ in increasing the HTLV-1 receptors on infected cells, such as Heparan Sulfate Proteoglycan (HSPG), Neuropilin-1 (NRP-1), and glucose transporter 1 (GLUT1). Therefore, these two proteins can facilitate the HTLV-1 attachment, binding, and fusion on APCs such as pDC and CD4+ T cells (20, 52). The last question is why the induced humoral response is not protective? A previous study declared that the active and protective immune response against retrovirus is mediated by cellular immunity, while the specific CTLs may have a key role in immune protection (43). The CFA and IFA adjuvant used in our study induced a predominantly Th2-biased response while the cell-mediated immune response needed activation of Th1. Therefore, humoral responses will be the prominent immune reaction against our recombinant protein (Figure 9). 

As mentioned before, TAX and HBZ are the two main proteins in the pathogenesis of HTLV-1 and have a proper immune identification facility. Therefore, they can be used for producing immune responses. As our study is the first attempt at creating the human Fc fusion HTLV-1 protein, the correct chemical structure in proper concentration is the first necessary step for immune response induction. Our results demonstrate that the Fc- TAX and HBZ recombinant protein can produce the monomeric and dimeric forms of fusion proteins in exact molecular weight (Figures 6 and 7). Moreover, our previous in silico examination proved the possibility of deigned proteins in *P. Pastoris *as a eukaryotic expression system (Figures 2 and 3).

Furthermore, the ability of these recombinant proteins to attach to CD 64 for presentation improvement in immune activation was observed by colocalization assay (Figure 8). We demonstrated that both recombinant proteins could produce the humoral immune response via specific antibody production (Figure 9). The uses of neutralizing antibodies were examined in previous studies. Begum et al. investigated the inhibitory binding properties of rat monoclonal antibody LAT-27 against 37 expressing clones of gp46 and suggest it can be used as a proper target for immune inhibitory responses (53). These results were demonstrated by a future study in 2016; it revealed that Anti-HTLV-1 gp46 neutralizing monoclonal antibody (LAT-27) could have potential for passive immunization against HTLV infection in both vertical and horizontal spread HTLV-1 (54). These results support the use of passive immune responses to treat HTLV-1 infection. The point around TAX and HBZ protein application for specific therapeutic antibody production is that these viral elements are expressed in a pathogenic state of infection. Therefore, any responses against them could bring more value to treatment. Our previous study demonstrates the indirect correlation of TAX, HBZ expression, and proviral load by survival period of patients. Therefore, targeting these HTLV-1 proteins can inhibit the progression of the associated diseases (17). 

The present study has some limitations, such as the sample size of rabbits in challenging assays for inducing protective immune responses. In HBZ: hFcγ, one animal expired. Concerning the specific anti-Tax and anti- HBZ neutralization properties, in the future, the neutralization assays must be performed in *in vivo* conditions. The challenging assay should be performed in sheep, which are more susceptible to HTLV-1 with both recombinant proteins.

## Conclusion

The results suggest that recombinant TAX: hFcγ1 and HBZ: hFcγ1 proteins can induce proper specific humoral immune responses. Both specific antibodies had the capacity for HTLV-1 neutralization *in vitro*. Therefore, they could be novel passive immunotherapy sources for HTLV-1-associated diseases, such as ATLL and HAM/TSP. Furthermore, these antibodies should be useful for making serological kits, such as ELISA, for HTLV-1 diagnosis.

On the other hand, challenging assays showed that none of these recombinant Fc fusion proteins could act as an effective vaccine to induce protective immunity.

## Authors’ Contributions

All authors contributed to the study conception. This study was designed by SAR. Data collection and analysis were performed by MMA, SAR, AM, SS, and HR. The *in silico* analysis was done by AAA and MMA. The animal practices were done by AAA and MMA. The first draft of the manuscript was written by MMA, and all authors commented on previous versions of the manuscript. The first draft was edited by SA R. All authors read and approved the final manuscript.

## Conflicts of Interest

The authors declare that they have no competing interest.
